# Longitudinal prediction of positive and negative mental health in Germany, Russia, and China

**DOI:** 10.1371/journal.pone.0234997

**Published:** 2020-06-23

**Authors:** Jürgen Margraf, Xiao Chi Zhang, Kristen L. Lavallee, Silvia Schneider

**Affiliations:** Mental Health Research and Treatment Center, Ruhr-Universität Bochum, Bochum, Germany; University of the Witwatersrand, SOUTH AFRICA

## Abstract

The present study examines a broad range of longitudinal predictors of dimensional positive mental health (PMH) and negative mental health (NMH), using data from the BOOM study. Participants were drawn from university student samples in Germany (1,608), Russia (677) and China (12,057). A structural equation model was conducted with four groups of predictors and PMH/NMH as criteria (outcomes). Five of the six salutogenic predictors were predictive of both positive mental health (positively) and negative mental health, as measured by depression (negatively). Pathogenic predictors anxiety and depression were related to future depression, but not to positive mental health. Stress at baseline was related to both future positive mental health (negatively) and future depression (positively). Being male in this study was associated with increased future depression. Results hold across Germany, Russia, and China. Results support the conceptualization of positive and negative mental health as related, but independent dimensions across three cultures.

## Introduction

Historically, mental health has been defined defacto as the absence of psychopathology [1–3]. However, this view is now recognized increasingly as overly simplistic, with positive mental health being a distinct and important construct for understanding the human condition [1, 2]. With the rise of positive psychology, there is an increased interest in facilitating human flourishing and thriving, not just establishing a neutral state of mind [4]. As awareness of a complete picture of mental health grows [5, 6], the traditional unidimensional model of mental disease is no longer sufficient. As positive mental health (PMH) and mental health problems can present simultaneously, they best viewed as separate interdependent dimensions [1, 2, 7], and should both be taken into account in research on mental health [8]. Still, most studies in clinical psychology and psychiatry continue to exclusively focus on negative aspects of mental health.

In addition to the importance of examining positive mental health, substantial progress in the understanding of the complex causes and correlates of mental health should involve examination of both salutogenic and pathogenic factors [9, 10], in the domains of not only psychological, but also biological and social factors. In the past, true interdisciplinary approaches have been rare exceptions; however the relevance of biological factors has been well established in the past two decades. In addition, social factors, both on the individual level (e.g. socioeconomic status, social mobility, personal ties) and the aggregate level (e.g. community health, neighborhood deprivation, inequality) show close links with physical and mental health [11–14]. Some important population parameters have been changing over the last century, with corresponding changes in mental health, including family relationships, social connectedness, expectations for having children, single parenting as risk for poverty and social deprivation, and mass media exposure [15] As a consequence, the settings in which people develop have changed dramatically within only a few decades. Within such work, longitudinal studies represent the gold standard in mental health prediction research (Ghaderi & Scott, 2001), with large-scale such research being ideal but underrealized (e.g. Moffit *et al*., 2007; Sihvola *et al*., 2010; Wang, 2004; Beard *et al*., 2008), especially for positive dimensions of mental health [3].

Pathogenic factors include predispositions (preexisting genetic, somatic, psychological or social characteristics that make the occurrence of a mental health problem possible or more probable), triggering conditions (negative experiences or stressors that trigger the primary occurrence of a mental health problem) [6, 16–18], and maintaining factors (dysfunctional reactions, such as avoidance, or cognitive distortions, or ongoing stressors that can prevent resolution of mental health problems and make them chronic [19–21]). Examples of salutogenic, protective factors are emotional stability, social support, sustainable relationships, sense of coherence and control, problem solving skills, and social and communication skills. These factors may also be involved in the generation of health, as they enable individuals to realize their potential, to cope with the normal stresses of life, to work productively and fruitfully, and to be able to make a contribution their communities [22]. That is, salutogenic factors may act upon all three classes of pathogenic factors to counteract or balance them [7, 23]. The present study is particularly focused on salutogenic factors and their prediction of positive mental health in contrast to pathological mental states.

### Psychological variables

Several psychological variables have particular value as predictors of mental health outcomes in previous research, and are also able to be reliably and validly assessed in the context of a large-scale surveys across cultures [7, 24–35]. These include traits such as resilience and personal values, as well as states such as current happiness, life satisfaction, sense of coherence, and the desire to have children [13, 24, 36, 37]. Studies have implicated salutogenic factors, such as sense of coherence (Eriksson & Lindström, 2007), gratitude and agreeableness (Wood *et al*., 2009), in the development of mental health and life satisfaction (Trumpf *et al*., 2009; Vriends *et al*., 2007). Resilience in particular is positively related to positive mental health (Haddadi & Besharat, 2010), and negatively associated with depressive symptoms (Brunwasser, Gillham & Kim, 2009).

Despite the importance of salutogenic factors, most studies still focus on pathogenic predictors and a limited number of specific disorders or negative dimensions, despite high comorbidity (Kessler *et al*., 2005; WHO, 2004), highlighting the need for more large-scale comprehensive, inclusive research studies. As many people experiencing mental health issues experience more than one type, and as many negative predictors predict more than one disorder type (Beard *et al*., 2007), it appears important to examine the fundamental processes underlying broad-based positive and negative mental health, and not just one or two narrow subtypes at a time. One recent large-scale longitudinal study of broad based mental disorders found that pathogenic predictors predict incidence and relapse of mental disorder diagnoses, while salutogenic factors predict remission over time [36]. Across the board, there is a need for more studies on salutogenic psychological predictors of positive mental health over time.

### Biological variables

There is strong evidence elucidating a bidirectional relationship between mental and physical health, until now primarily focused on health problems and mental disorder (Kolappa et al. 2013). More broadly, it is generally accepted that many physical conditions are associated with serious mental disorders, but the exact nature of these relationships is still unclear and further research beyond the so far dominant cross-sectional designs is called for [38]. Biological variables include physical health [13, 39], body mass index (BMI) [40, 41], sex [42, 43], and age [13, 44–46], are important contributors to mental health and can be assessed easily within the framework of a large-scale survey.

### Social variables

People with low SES, as measured by income, tend to have lower levels of positive mental health [47, 48]. Further, social support and social rhythm as associated with positive mental health outcomes [13, 18, 24, 35, 36]. The role of perceived social support as a protective factor for mental health problems is well-established [49]. Aside from reducing mental illness incidence, perceived social support also reduces general stress in women and men [50] and has positive effects on somatic health [51]. Social rhythm refers to the regularity with which one engages in social activities throughout the week, and has established links with bipolar disorder, as well as some links with depression and anxiety [52]. Just as daily biological patterns, such as circadian rhythm, temperature fluctuations, and cortisol levels, are integral to good mental health, with disruptions associated with depression [53], so it appears are rhythmic social and behavioral patterns, for example in mealtimes, bedtimes, and patterns of social interaction [35, 54, 55]. While next to no research exists on plans for fertility and mental health, fertility wish may be a potential sign of optimism about the future, and thus a social indicator that may be related to positive mental health outcomes. Indeed, in western societies, those who do have children live longer than those who don’t [56]. Having children can provide older adults with important social support, and plans to have them may indicate a certain level of optimism about the world those children will encounter in the future.

With respect to negative mental health, a meta-analysis of 51 population-based surveys found that adults in the lowest socioeconomic position (SES) have an increased risk of developing depression, with an odds ratio of 1.81 as compared to those in the highest position [57]. Also, recent results from a national survey show that the 12-month prevalence rate for mood disorders is more than twice as high for low (14%) than for high SES (6.3%) [13]. Further, this social class gradation is present across the lifespan [58, 59].

### Cross-cultural approach

Despite the increased recognition of the importance of positive factors, so far, most studies into positive factors have been conducted in Western nations, limiting the generalizability of the findings. Depth of inquiry into of the nature of positive protective constructs across cultures is long overdue. Psychological, biological and even sociological theories of mental health typically strive for universal validity, and the claim to be transcultural (i.e., universal). However, social factors and cultural background are widely recognized as a potentially important influences in mental health [11–14, 37]. However, we cannot continue to postulate universal validity for psychological theories that have not been tested or may even not be amenable to testing across cultural boundaries. For our theories to be truly transcultural, they must first be studied cross-culturally [60].

### Present study

The present study is the primary outcome study in the the “Bochum Optimism and Mental Health (BOOM) Studies” [61], which aim to enhance integrated knowledge of the causes and consequences of positive mental health and mental health problems cross-culturally and over time. The present analysis, specifically, is a large-scale, cross-cultural, multi-national, and longitudinal investigation into primarily salutogenic factors contributing to both positive and negative mental health outcomes, as measured by positive mental health and depression. It is an attempt to contribute to a comprehensive picture of the etiology of mental health. This study examines the following positive constructs thought to be relevant to mental health (positive mental health) and illness (depression, stress, anxiety): resilience, social support, social rhythm, family affluence, physical health and expectations for fertility. Finally, dimensional measures of mental health will be examined, rather than solely categorical measures. Factors will be examined across two time points, and across three countries: Germany, China, and Russia. We limited the number of predictors for methodological reasons [62]. We hypothesized that mental health will be predicted by the balance between positive (protective, salutogenic) and negative (pathogenic) psychological, biological and social factors and that these relationships will have an influence on mental health longitudinally [7, 36].

## Method

### Procedure

The present study utilizes a subset of data from the Bochum Optimism and Mental Health (BOOM) study, a large-scale, cross-cultural, longitudinal investigation of risk and protective factors in mental health [37, 61]. A comprehensive overview of the full study design, aims, measures, and participants is provided elsewhere [61]. The Ethics Committee of the Faculty of Psychology of the Ruhr University Bochum approved the study. Approval to administer the questionnaires was granted by the Faculty of Psychology at Ruhr University Bochum on May 12, 2011 and renewed on September 2013. The approvals for the German site were communicated to the participating Chinese and Russian Universities who acknowledged these approvals. Data were collected between 2011 and 2016 Participants in the present study were recruited via the internet (German, Russian, and Chinese) and paper mailings (Chinese). The period of time between the first and second time points was about 17 months for each participant. All participants were informed that their answers would be collected pseudonymised, which means that a code is used for anonymisation. This makes it possible to relate individuals‘ answers to one another across different data records, while maintaining the person’s anonymity. Only the project supervisors have access to these codes. Participants provided implicit consent by choosing to continue with the questionnaires after reading the informed consent statement.

German data collection at Ruhr University Bochum was via an online portal, with data collection beginning in 2011. The Ethics Committee of the Faculty of Psychology of Ruhr University Bochum approved the study on May 12, 2011 and renewed on October 2012. The German sample at baseline consisted of 7,890 students from Ruhr University Bochum from 2012 to 2015. In the first follow-up study, 1,608 students participated again. The link was sent to all students enrolled at Ruhr University Bochum in 2012 and sent only to freshmen at from 2013 to 2015. Students were offered the opportunity to take part in a draw for a gift coupon (20 euro) or a tablet computer.

The Russian sample consisted of 3,745 students from Lomonossov University Moscow, University of Voronesh, and University of Orenburg in 2013. In the follow-up study in 2014, 677 students participated again. Participants were recruited via an invitation letter. Data were gathered by online and paper-and-pencil questionnaires administered in a group testing session. Participants received no financial compensation. +

In China, as the data were anonymized from the very beginning of data collection, no statement by an institutional board/ethics committee was required for China. The original Chinese sample at baseline consisted of 13,581 university students from Capital Normal University Beijing, Hebei United University, Shanghai Normal University, Guizhou Finance and Economics University, and Nanjing University with baseline data collected from 2012 to 2013. Of those, 12,744 students participated again in the first follow-up study between 2013 to 2014. Most of them were freshmen at baseline survey.

### Participants

University student participants with data at baseline and one-year follow-up were included from three countries: Germany (1,608), Russia (677) and China (12,057). Representativeness of the students samples as compared with the adult residential populations in the three countries was assessed based on the register-assisted census data from 2011 regarding age, gender and education, was ensured via systematized sampling procedures.

#### Germany

The German sample consisted of 7,890 (1,608 had data at follow-up) student participants recruited from Ruhr University Bochum. Students were assessed via online survey. German students were recruited by an e-mailed invitation with a link leading to an online questionnaire. The link was sent to all students enrolled at Ruhr University Bochum. They were offered an incentive to take part in a drawing for a gift certificate or a tablet computer.

#### China

As the data were anonymized from the very beginning of data collection, no statement by an institutional board/ethics committee was required to collect data in China. The Chinese sample consisted of 13,581 (12,057 had data at both time points) university students from the Capital Normal University Beijing, the Hebei United University, Shanghai Normal University, Guizhou Finance and Economics University, and Nanjing University. Participants, mainly freshmen, were recruited during their first study month via an invitation by mail. The response rate was 94.5%. Data were gathered by an online or a paper-pencil questionnaire administered in a group testing session. Participants received 10 RenMinBi (approximately 1.3 Euros) upon returning the questionnaire.

#### Russia

As the data were anonymized from the very beginning of data collection, no statement by an institutional board/ethics committee was required to collect data in Russia. The Russian sample consisted of 4,001 (677 had data at both time points in the present analyses) students from Lomonossov University Moscow, the University of Voronesh, and the University of Orenburg. Participants were recruited via an invitation letter. The response rate was 95.3%. Data was gathered by means of online and paper-pencil questionnaires, administered in a group testing session. Participants received no financial compensation.

### Measures

#### Overview

As far as possible, established brief standard instruments such as the Depression Anxiety Stress Scales (e.g., DASS 21) were used to measure the constructs of interest. For all questionnaires used in the analysis, validated German versions exist. Russian and Chinese versions of the measures were developed when needed, by using the customary translation-back-translation method as recommended [63]. In cases of discrepancies, this procedure was repeated by the study team until complete agreement was achieved. Measures can be grouped according to the overall design of the research program. Table 2 provides an overview as well as correlations.

#### General outcomes

*Positive mental health*. The 9-item PMH-scale was developed in order to provide a brief, uni-dimensional and person-centered instrument to assess positive mental health [7]. The concept of positive mental health combines mainly emotional, but also psychological and social aspects of well-being into a single general construct [7]. People who are mentally healthy tend to have stable relationships, view their lives as having purpose and direction, experience more positive affect, and are more likely to be self-accepting [64]. Psychometric testing confirmed the scale to be a unidimensional self-report instrument with high internal consistency, good retest-reliability, scalar invariance across samples and over time, good convergent and discriminant validity as well as sensitivity to therapeutic change in a series samples from very different backgrounds [7]. Participants respond to statements such as *“I am often carefree and in good spirits*, *I enjoy my life*, *I manage well to fulfill my needs*, *I am in good physical and emotional condition”* on a 4-point likert scale ranging from 1 (do not agree) to 4 (agree). Item scores are combined into a sum score with higher scores indicating higher positive mental health. The measurement invariance of positive mental health is established as full strong (Bieda, Hirschfeld, Schoenfeld, Brailovskaia, & Zhang, 2017). Cronbach’s alphas at baseline were .916 (Germany), .891 (China), and .867 (Russia).

*Depression*, *anxiety and stress*. Negative mental health was assessed using the widely- used Depression Anxiety Stress Scales (DASS-21) [65]. This short form of the DASS-42 [66] assesses a broad range of psychological distress symptoms. It is composed of three 7-item subscales for depressive, anxiety and stress symptoms over the past week. The subscales may serve as outcome measures and as screening and monitoring instruments [67–69]. Items are rated on a 4-point likert scale from 0 (did not apply to me at all) to 3 (applied to me very much or most of the time). Responses can be averaged within subscale or across all three for a total item score. Psychometric properties are well established in both clinical and non-clinical samples [65, 69] and are comparable for the short and long versions [70, 71]. The measurement invariance of these three scales are established as full strong among the German, Russian, and Chinese students samples. In the present study, Cronbach’s alphas at baseline for depression were .886 (Germany), .776 (China), and .794 (Russia). Alphas for anxiety were .774 (Germany), .737 (China), and .775 (Russia). Alphas for stress were .853 (Germany), .771 (China), and .774 (Russia).

#### Predictors

*Resilience*. Psychosocial stress resilience was assessed with an 11-item short version of the Wagnild and Young Resilience Scale (RS-14; 100; RS-11; 102) [72]. Participants responded to items such as “I usually manage one way or another” on a scale ranging from 1 (I disagree) to 7 (I agree). The RS-11 demonstrated good reliability and convergent validity in a German sample [73]. The measurement invariance of resilience is established as partial strong (Bieda, Hirschfeld, Schoenfeld, Brailovskaia, & Zhang, 2017). Cronbach’s alphas at baseline were .891 (Germany), .793 (China), and .776 (Russia).

*Social support*. Social support was assessed using the 14-item Questionnaire- Social Support measuring perceived and/or anticipated social support (F-SozU K-14) [74]. Participants indicated agreement with statements such as “I experience a lot of understanding and security from others” on a 5-point Likert scale ranging from 1 (*not true)* to 5 (*true*). In a German population, this unidimensional measure showed excellent Cronbach`s α and good convergent and discriminant validity [74]. In cross-cultural research, Nover (2012) [75] tested a long version of the Questionnaire- Social Support (F-SozU-22) [76] among pupils from Germany, Luxembourg and Spain, finding partial weak measurement invariance for the three cultural groups. The measurement invariance of the F-SozU K-14 is established as partial strong [77]. Cronbach’s alphas at baseline were .935 (Germany), .948 (China), and .929 (Russia).

*Social rhythm*. Social rhythm was assessed using the Brief Social Rhythm Scale (BSRS) [35]. This scale consists of ten items, which assess the irregularity with which participants engage in basic daily activities during the workweek and on the weekend. The BSRS assesses waking and bedtimes and breakfast and dinner mealtimes. It also assesses the regularity of time spent with others at work/school and during free time. Participants are asked to rate the general regularity of each activity in their lives in general using a scale ranging from 1 (very regularly) to 6 (very irregularly), with high mean scores indicating high irregularity. This measure can be administered at a single time point, rather than requiring a week of daily data to score. Summary scores are the sum across all 10 items. The BSRS shows a slight positive skewed distribution. It is reliable, distinguishes among categories of mental health and detects relationships with physical and mental health, and is especially useful in large-scale or screening studies, where participant time is limited [35]. In the German representative telephone data, item-total correlations ranged from r = .25 to r = .54. Test-retest-reliability in a subsample study of 1294 people from Germany from the BOOM study who took the measure online or in paper and pencil format at time 1 and time 2 (4 weeks later) was r = .70. Cronbach’s alpha was α = .763 in Germany, .837 in China, and .775 in Russia. The measure was reversed scored in the present study so that high scores equal higher rhythmicity.

*Fertility wish*. The wish to have children (“Kinderwunsch” in German) was assessed using a single yes-no item asking “Do you want to have a child/children in the future?” Participants who already had a child, were asked “Do you want to have one more child / more children?” and responded indicating “no” or “yes” with how many children they wanted to have. Those answers were recoded into simple “no” or “yes.” In the Chinese and Russian samples, we only used data from people with no existing child. In the German sample, 840 of total 1558 responses to this question were from people who definitively had no existing child, and the rest may or may not have had a child already.

*Quality of health*. Overall current quality of health was assessed using the EuroQol (EQ-VAS) [78–80]. Participants rated current health status on a scale ranging from 0 (worst imaginable health) to 100 (best imaginable health). Validity of EQ-VAS is indicated by convergence with the the five dimensional version of the EuroQol (EuroQol 5D) with WHO-5 and known clinical groups across several countries.

#### Sociodemographic predictors

*Basic sociodemographic predictors*. Sex, age, and relational status were assessed via self-report.

*Family affluence*. To ensure sufficient comparability across vastly different countries, the Family Affluence Scale (FAS) [81] served as the main cross-cultural measure of socioeconomic circumstances. The FAS is, a four-item measure of family wealth, developed in the WHO Health Behavior in School-aged Children Study. Questions include (either with 2 or 3 response alternatives): “Does your family own a car, van or truck?”, “Do you have your own bed- room for yourself?”, “During the past 12 months, how many times did you travel away on holiday with your family?”, and “How many computers does your family own?”. The FAS total score is calculated by summing up the responses to these items. Convergent validity is established via correlations with the Gross National Product across 35 countries [81]. Cronbach’s alphas at baseline were .315 (Germany), .640 (China), and .379 (Russia).

### Statistical analyses

The scales fulfilled the minimum requirement for path comparisons in cross cultural studies: weak invariance. A series of structural equation models were conducted with all the above mentioned predictors at baseline and positive mental health and depression at follow-up as outcomes for each country separately to specify the relations between variables in this study. After that, a multi group analysis was carried out to examine the role of country. The baseline model (M1) with no constrains on the paths will be tested in the first step. Then the same model but with all paths being constrained as the same across the three countries (M2) will be tested. Model fit indices will be examined to further assess the model’s fit. The root mean square of approximation (RMSEA) will be interpreted as follows: values above 0.10 indicate unacceptable fit [82], and values in the range of 0.08 to 0.10 indicate mediocre fit, those between 0.05 and 0.08 indicate fair fit, and those less than 0.05 indicate close fit [83, 84]. The comparative fit index (CFI) [85] with values, which are greater than .90, indicates a good fit. Since equality constraints will mostly lead to decreases in fit indices and the χ2 difference test is highly sensitive in large samples (Oishi, 2007), the rule of ΔCFI not greater than 0.01 [86] is recommended for model comparison. All analyses were calculated with SPSS 23 and R version 3.4.2 with the Package “SEM”.

Missing values were generally between .2 to 2.6%, depending on the measure, with Social Rhythm having 13.3% due to an error omitting 2 questions for some participants (missing at random with no statistical correlation to data). This had no statistical relationship to the results, and therefore participants with questionnaires with missing data relevant to the analyses were deleted from the analyses that involved those questionnaires. Further, as assessment methods had an influence on the data, in all analyses the influence of assessment method was controlled [87]. Internal consistency is computed with Cronbach’s α coefficient. Cronbach’s *α* > 0.70 indicates acceptable, > 0.80 good, and > 0.90 excellent internal consistency [88]. Data are available in Supporting Information File 1.

## Results

### Descriptive statistics

Table 1 presents data on participant demographics and descriptive statistics for the predictors and outcomes at baseline.

**Table 1:** Descriptive statistics of socio-demographic variables and measures.

	China	Germany	Russia
	N = 12057	N = 1608	N = 677
Gender			
Female	7390	1028	281
Male	4514	580	353
Partner			
Single	1848	708	281
Single with Partner	10121	814	353
Fertility Wish			
Yes	10661	1359	613
No	1366	199	64
Mean (SD)			
Age in BL	19.63(1.66)	23.58(4.73)	19.63(2.16)
Family Affluence in BL	2.75(2.15)	3.91(1.79)	4.41(1.93)
Stress in BL	3.33(3.11)	7.23(4.68)	7.07(4.40)
Anxiety in BL	2.91(2.72)	3.15(3.44)	4.07(3.88)
Depression in BL	1.81(2.45)	4.35(4.38)	4.38(3.89)
Health state in BL	87.16(11)	77.18(19.63)	72.97(18.68)
Social support in BL	56.82(11.95)	60.03(10.13)	58.12(10.91)
Resilience in BL	58.50(8.42)	58.17(10.94)	59.91(8.21)
Postive mental health in BL	21.17(4.98)	18.45(5.69)	18.88(5.14)
Social rhythm in BL	43.64(7.93)	41.00(8.15)	31.78(8.81)
Postive mental health in FU	19.92(5.21)	18.18(5.89)	17.98(5.27)
Depression in FU	2.56(3.47)	4.36(4.50)	4.59(4.25)

### Correlations

The correlations among the psychological predictors are shown in Table 2. The salutogenic predictors (positive mental health, life satisfaction, self-efficacy) are generally positively correlated with each other. The baseline negative aspects of mental health, or pathogenic predictors (i.e., stress, anxiety, and depression) are positively correlated with each other as well. The salutogenic predictors generally correlated negatively with the pathogenic predictors. Global assessment of functioning correlated positively with the salutogenic, and negatively with the pathogenic predictors.

**Table 2:** The correlations among the psychological predictors within country.

	Control	Baseline pathogenic predictors			Baseline salutogenic predictors					Outcomes at follow-up	
	Family Affluence	Stress	Anxiety	Depression	Health state	Social support	Resilience	Positive mental health	Social rhythm	Positive mental health	Depression
**China**											
Family Affluence in BL	1										
Stress in BL	-.003	1									
Anxiety in BL	.005	.726**	1								
Depression in BL	-.019*	.662**	.671**	1							
Health state in BL	-.076**	-.360**	-.369**	-.378**	1						
Social support in BL	.153**	-.184**	-.179**	-.244**	.128**	1					
Resilience in BL	.054**	-.320**	-.325**	-.392**	.285**	.309**	1				
Postive mental health in BL	.073**	-.480**	-.453**	-.527**	.366**	.351**	.539**	1			
Social rhythm in BL	.107**	-.282**	-.269**	-.259**	.182**	.157**	.334**	.341**	1		
Postive mental health in FU	.068**	-.308**	-.283**	-.302**	.246**	.201**	.308**	.434**	.227**	1	
Depression in FU	-.034**	.323**	.309**	.352**	-.204**	-.155**	-.198**	-.284**	-.170**	-.443**	1
**Germany**											
Family Affluence in BL	1										
Stress in BL	-.112**	1									
Anxiety in BL	-.076**	.556**	1								
Depression in BL	-.116**	.594**	.605**	1							
Health state in BL	.037	-.323**	-.352**	-.351**	1						
Social support in BL	.114**	-.242**	-.298**	-.448**	.229**	1					
Resilience in BL	.036	-.235**	-.245**	-.397**	.237**	.307**	1				
Postive mental health in BL	.150**	-.532**	-.487**	-.694**	.431**	.528**	.494**	1			
Social rhythm in BL	.101**	-.238**	-.199**	-.275**	.174**	.237**	.216**	.299**	1		
Postive mental health in FU	.120**	-.425**	-.371**	-.528**	.339**	.422**	.394**	.683**	.279**	1	
Depression in FU	-.098**	.407**	.406**	.576**	-.257**	-.346**	-.270**	-.498**	-.237**	-.673**	1
**Russia**											
Family Affluence in BL	1										
Stress in BL	-.102**	1									
Anxiety in BL	-.114**	.683**	1								
Depression in BL	-.079*	.613**	.618**	1							
Health state in BL	.125**	-.347**	-.344**	-.387**	1						
Social support in BL	.106**	-.204**	-.170**	-.297**	.243**	1					
Resilience in BL	.123**	-.260**	-.217**	-.406**	.322**	.313**	1				
Postive mental health in BL	.155**	-.434**	-.386**	-.585**	.412**	.451**	.506**	1			
Social rhythm in BL	-.047	.018	.012	-.023	.108*	-.076	.115**	.063	1		
Postive mental health in FU	.080*	-.256**	-.206**	-.296**	.256**	.198**	.265**	.354**	-.061	1	
Depression in FU	-.05	.223**	.184**	.258**	-.177**	-.178**	-.176**	-.274**	.127**	-.520**	1

* p < .05. two-tailed.

** p < .01. two-tailed.

*** p < .001. two-tailed.

### Structural equation model

**Fig 1:**
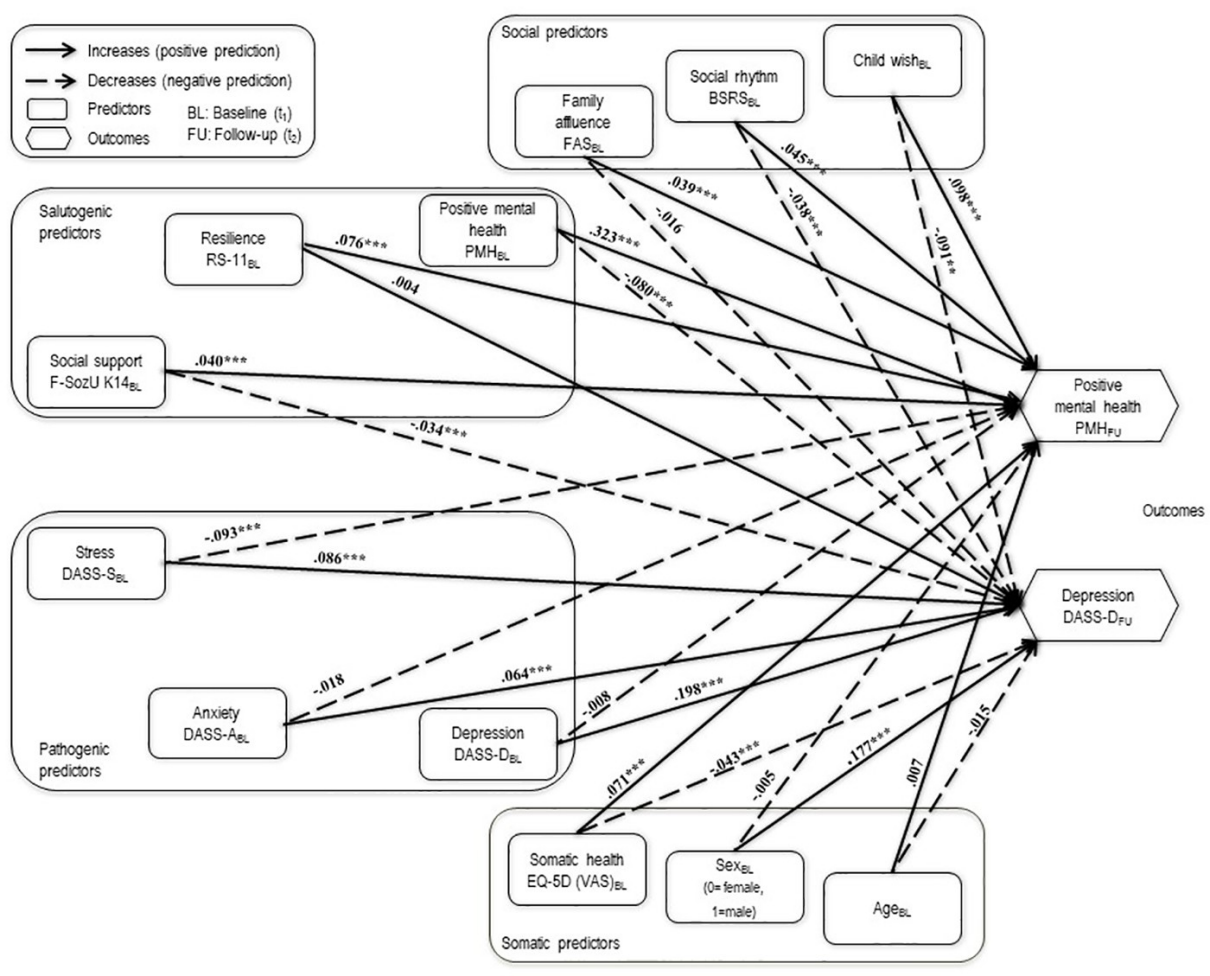
Model with all path coefficients constrained to be equal across China, Germany, and Russia.

Table 3 provides the model fit for the unconstrained model (M1) as well as the constrained model (M2). In M1, each country’s regression weights were estimated separately. In M2, the regressions weights were constrained to be equal across countries. Fig 1 displays the constrained model (M2). The same unconstrained model (M1) was tested in each country and model fits well according to the fit indices. M1 was then calculated for all three countries together as a baseline model, also resulting in good fit, with CFI = .916, RMSEA = .087, and SRMR = .072. After the regression coefficients were held equal across three countries (M2), the model still provided an appropriate fit to the data, and ΔCFI did not exceed .01. Thus, the model fit can be considered equal to the M1 [89]. Standardized regression coefficients of the single equations from the unconstrained model (M1) for each country as well as from the constrained Model (M2) are presented in Table 4.

**Table 3:** Fit indices for the unconstrained and constrained models.

	χ^2^	df	CFI	RMSEA	SRMR	Δχ^2^	Δdf	p	ΔCFI
M1									
China	2539,193	38	.917	.085	.068				
Germany	538,753	38	.910	.100	.101				
Russia	168,245	38	.908	.083	.071				
Baseline model	3246,191	114	.916	.087	.072				
M2	3455,915	162	.911	.075	.074	209,724	48	< .001	.005

**Table 4:** Standardized regressions-coefficients from structural equation models.

Outcome		M1			M2
		China	Germany	Russia	
Depression_FU	Explained variance	.145	.317	.128	
	Gender	.106***	.062	.091	.177***
	Age	-.012	-.001	-.083	-.015
	Family Affluence in BL	-.015	-.007	.012	-.016
	Stress in BL	.100***	.046	.056	.086***
	Anxiety in BL	.065***	.067*	-.007	.064***
	Depression in BL	.166***	.353***	.075	.198***
	Health state in BL	-.045***	-.027	-.081	-.043***
	Social support in BL	-.022*	-.077**	-.010	-.034***
	Resilience in BL	-.001	.001	.012	.004
	Postive mental health in BL	-.068***	-.118***	-.211***	-.080***
	Social rhythm in BL	-.046***	-.051*	.118**	-.039***
	Fertilitywish_BL	-.036***	.074	-.258	-.091**
PMH_FU	Explained variance	.199	.436	.167	
	Gender	.002	-.002	-.024	-.005
	Age	.015	-.030	.053	.007
	Family Affluence in BL	.047***	.011	-.012	.039***
	Stress in BL	-.091***	-.082**	-.092	-.093***
	Anxiety in BL	-.024	-.008	.036	-.018
	Depression in BL	-.002	-.010	-.022	-.008
	Health state in BL	.069***	.051*	.132**	.071***
	Social support in BL	.034*	.074**	-.002	.040***
	Resilience in BL	.073***	.079**	.099*	.076***
	Postive mental health in BL	.295***	.496***	.212***	.323***
	Social rhythm in BL	.056***	.040	-.095*	.046***
	Fertilitywish_BL	.022*	.083	.268	.098***

* p < .05.

** p < .01.

*** p < .001.

The results of the model indicated that nearly all of the six salutogenic predictors were predictive of both positive mental health (positively) and negative mental health, as measured by depression (negatively). The only salutogenic factor not related to future depression was resilience. Pathogenic predictors anxiety and depression were related to future depression, but not to positive mental health. Stress at baseline was related to both future positive mental health (negatively) and future depression (positively). The control variable family affluence was positively related to positive mental health. Being male in this study was associated with increased future depression.

## Discussion

To our knowledge, this is a unique longitudinal, prospective study of its size in its examination of the relationship between positive and negative mental health cross-culturally, with such a wide range of psychological salutogenic predictors. The present study extends earlier findings by providing evidence that salutogenic factors not only predict future positive and negative mental health, but may be more likely to predict both positive and negative future mental health than pathogenic variables. The findings speak to the importance of resilience, and extend past findings beyond the typical North American studies, across Euro and Asian cultures, to Germany, Russia, and China. Results indicated that in all three countries, all of the six salutogenic predictors, including somatic health, social support, resilience, positive mental health, social rhythm regularity, and fertility wish, were predictive of both positive mental health (positively) and negative mental health, as measured by depression (negatively). The only salutogenic factor not related to future depression was resilience. Pathogenic predictors anxiety and depression were related to future depression, but, contrary to our predictions, these factors were not important in predicting future positive mental health once baseline salutogenic factors were taken into account. Stress at baseline was indeed related to both future positive mental health (negatively) and future depression (positively). As predicted, and in line with past research, family affluence was positively related to positive mental health [13, 57]. Being male in this study was associated with increased future depression. This result is in contrast to prior research indicating that females typically report greater depression and lower overall internalizing mental health [13, 42]. Notably, many of the effects were small. The large sample size allowed for such a complex model to detect small, but meaningful and significant effects.

The model fit was acceptable when the model was constrained to be equal across cultures, indicating that in general, the variables were predictive in a similar way across China, Germany, and Russia. Interestingly, and unique to this study, the desire to have a child was related to increased well being. It would be interesting to examine this factor in the future as a sign of optimism. Further, it was similarly related to well-being in all three countries. China has had fertility limiting policies in place, but have been relaxing them in recent years, and since before the current data were collected. It is not expected that state attempts to limit fertility would have had a large moderating impact on the influence of fertility desire on well-being, as the relationship from fertility desire to later depression or positive mental health did not display cross-cultural differences (that is, the constrained model was not significantly different from the unconstrained model).

The current findings point to the importance of positive mental health as a dimension in its own right, rather than simply an absence of mental health problems. Positive mental health is indeed a unique predictor not only of future positive mental health, but also of future mental health problems, above and beyond the predictive value of baseline negative mental health. However, it would be an oversimplification to aim toward positive feelings and away from negative feelings without taking context into account. As some authors have recently pointed out, feelings of sadness are healthy and normal in some circumstances, and feelings of positivity can be inappropriate and maladaptive under certain conditions. Life is a balance of positive and negative, both reactions can have their place as appropriate responses to life circumstances, and neither reaction is necessarily pathological [90, 91]. Future researchers are encouraged to examine both positive and negative emotions as not simply ends in themselves but as reactions to a broader context that can themselves be either healthy or unhealthy responses. Positive emotions (i.e., happiness) do not necessarily have to be the ultimate aim. Rather, a focus on euthymia (tranquility or calm), the wisdom that comes from challenge, and mastery of life tasks may be more attainable or even appropriate aims [91].

This study has a number of strengths, including the large sample size, its thorough assessments using standardized instruments and follow-up interview. It also examines relationships among the factors outside of the typical North American sample, extending knowledge and comparing models cross-culturally, across Germany, Russia, and China. Despite its strengths, the present study also has limitations. First, the present study does not compare results to a traditional standard-bearer culture in Western psychological research: the USA. Though comparing results to the US should not be the litmus test of a good study, it would be interesting to see if relationships are the same there, and elsewhere. Second, while the present study was comprehensive, it was not exhaustive. A more exhaustive study would have included more pathogenic variables, such a problematic personality factors. Third, while the model provided a good fit to the data, the effects were small, implying that a more complete picture would need to include additional factors. The pathways to mental health are many and complex. This model provides only some of the whole picture. Fifth, the study used university student samples, which are not representative of the population, and thus generalizability is limited. Sixth, the study has vastly different dropout rates for Chinese (lower dropout) versus German (higher dropout) and Russian (highest dropout rates) participants. In Germany and Russia, the participants were assessed at baseline in various semesters, while in China the participants were primarily assessed at baseline during their first semester, which made them more able to join in the follow-up studies. Further, in Germany the participants were all from the same university, but in Russia they were from different universities under the management of different contact persons (some were no longer in the research area after one year.). Thus, the differences in drop-out rates were likely caused by outside conditions rather than by the participants themselves, and are therefore not thought to have an impact on results. Seventh, the present study indicated higher positive mental health and lower negative mental health for Chinese participants on some measures. Unfortunately, measures of social desireable responding were not included in this study, so we cannot know if those differences were true cultural differences, or may have been due to potential cultural differences in socially desirable responding. Eighth, the present study examined the effects of predictors on later positive and negative mental health, but did not examine the potential reciprocal effects of depression and positive metnal health on some of the predictors. For example, it may be that people with depression are more able to uphold daily social rhythms and routines over time, and that the longitudinal effects are actualy reciprocal rather than unidirectional. Finally, the study lacked clinical sub-samples and the time period studied was relatively short for longitudinal studies, which may partially explain the generally small relationships in the findings.

In sum, positive mental health is predictive of both positive and negative mental health aspects over time, while negative mental health is primariliy predictive of negative mental health, but not positive mental health, with the exception of stress, which predicts both positive and negative mental health over time. These results hold across Germany, Russia, and China. Positive mental health is not simply an absence of mental health problems, but rather is predictive independently. Positive and negative mental health should be viewed as related, but independent dimensions. Positive mental health and salutogenic predictors should be included by default in future studies of the prediction of mental health and disorder, and should be considered more thoroughly as a target for intervention in both alleviating mental disorder and contributing to human flourishing [92].

## Supporting information

S1 Appendix (DOCX)

S1 Data (SAV)
