# Supplementary material for: Longitudinal prediction of positive and negative mental health in Germany, Russia, and China
Source: PLoS One. 2020 Jun 23;15(6):e0234997. doi: 10.1371/journal.pone.0234997 (PMC7310683; doi:10.1371/journal.pone.0234997)
Supplement: S1 Appendix — (DOCX) [file pone.0234997.s001.docx]

Data transparency statement

This work is part of The BOOM Studies, a multi-national, longitudinal research study examining mental health across cultures and time. Other publications have arisen from this study, but do not replicate the analyses conducted in this paper.
